# Transcriptomics identifies blunted immunomodulatory effects of vitamin D in people with multiple sclerosis

**DOI:** 10.1038/s41598-024-51779-0

**Published:** 2024-01-16

**Authors:** Wei Z. Yeh, Rodney Lea, Jim Stankovich, Sandeep Sampangi, Louise Laverick, Anneke Van der Walt, Vilija Jokubaitis, Melissa Gresle, Helmut Butzkueven

**Affiliations:** 1 Department of Neuroscience, Central Clinical School, Monash University, Alfred Centre, Level 6, 99 Commercial Rd, Melbourne, VIC 3004 Australia; 2 Department of Neurology, Alfred Health, Melbourne, VIC Australia; 3 School of Biomedical Sciences and Pharmacy, University of Newcastle, Newcastle, Australia; 4 Centre for Genomics and Personalised Health, School of Biomedical Sciences, Queensland University of Technology, Brisbane, Australia; 5 Department of Medicine, University of Melbourne, Melbourne, VIC Australia

**Keywords:** Gene expression, Autoimmunity, Gene regulation in immune cells, Autoimmune diseases, Neuroimmunology, Neuroimmunology, Multiple sclerosis, Multiple sclerosis, Multiple sclerosis

## Abstract

Vitamin D deficiency is a risk factor for developing multiple sclerosis (MS). However, the immune effects of vitamin D in people with MS are not well understood. We analyzed transcriptomic datasets generated by RNA sequencing of immune cell subsets (CD4^+^, CD8^+^ T cells, B cells, monocytes) from 33 healthy controls and 33 untreated MS cases. We utilized a traditional bioinformatic pipeline and weighted gene co-expression network analysis (WGCNA) to determine genes and pathways correlated with endogenous vitamin D. In controls, CD4^+^ and CD8^+^ T cells had 1079 and 1188 genes, respectively, whose expressions were correlated with plasma 25-hydroxyvitamin D level (*P* < 0.05). Functional enrichment analysis identified association with TNF-alpha and MAPK signaling. In CD4^+^ T cells of controls, vitamin D level was associated with expression levels of several genes proximal to multiple sclerosis risk loci (*P* = 0.01). Genes differentially associated with endogenous vitamin D by case–control status were enriched in TNF-alpha signaling via NF-κB. WGCNA suggested a blunted response to vitamin D in cases relative to controls. Collectively, our findings provide further evidence for the immune effects of vitamin D, and demonstrate a differential immune response to vitamin D in cases relative to controls, highlighting a possible mechanism contributing to MS pathophysiology.

## Introduction

Vitamin D is a secosteroid hormone initially recognized for its important role in skeletal health. The main sources of vitamin D are ultraviolet B-induced synthesis in the skin, dietary intake or supplementation. Vitamin D is hydroxylated to 25-hydroxy-vitamin D (25(OH)D), and then again to 1,25-dihydroxyvitamin D which is its active form and also known as calcitriol. In target cells, calcitriol binds to the vitamin D receptor (VDR) and heterodimerizes with retinoid X receptor (RXR). This complex then binds to vitamin D response elements of the genome to regulate gene expression^1^. It is now recognized that multiple extraskeletal cell types express VDR and enzymes required to activate vitamin D. These include both innate and adaptive immune cells^2–4^. In vitro studies of immune cells cultured with calcitriol, albeit at supraphysiologic concentrations, have shown an enhancement of pathogen clearance in the innate arm and an immunoregulatory effect in the adaptive arm of the immune system^5–7^. The few human in vivo vitamin D supplementation studies that have examined peripheral immune cell responses showed reductions in proinflammatory IL-17-producing T cell numbers, and increase in regulatory T (Treg) cells, in support of an anti-inflammatory effect^8,9^.

Vitamin D deficiency is implicated in the development of a number of autoimmune diseases, including multiple sclerosis (MS)^10^. However, the specific mechanisms that lead to elevated MS disease risk are not well understood. Lymphocytes isolated from patients with MS develop a tolerogenic phenotype in response to calcitriol in culture^11,12^. However, several studies suggest differences in response when compared to cultured immune cells from healthy controls. These findings include a greater reduction in IL-17 secretion by activated T cells of healthy controls compared to MS cases^11^, and an increase in CD25 expression on CD46-stimulated T cells from healthy controls but not in those from MS cases^12^. Further, randomized-controlled trials of vitamin D supplementation in people with MS have not shown conclusive benefit on disease activity^13^. Although, intriguingly, the largest such study showed a significant reduction in MS lesions detected by magnetic resonance imaging among those assigned to vitamin D as add-on to MS immunomodulatory treatment of interferon-beta-1a^14^. Whilst vitamin D plausibly has therapeutic potential in diseases such as MS, its role in MS pathophysiology remains incompletely understood. Differences in vitamin D response between those with and without MS, and within different immune cell subsets, also remain open to further investigation.

**Figure 1:**
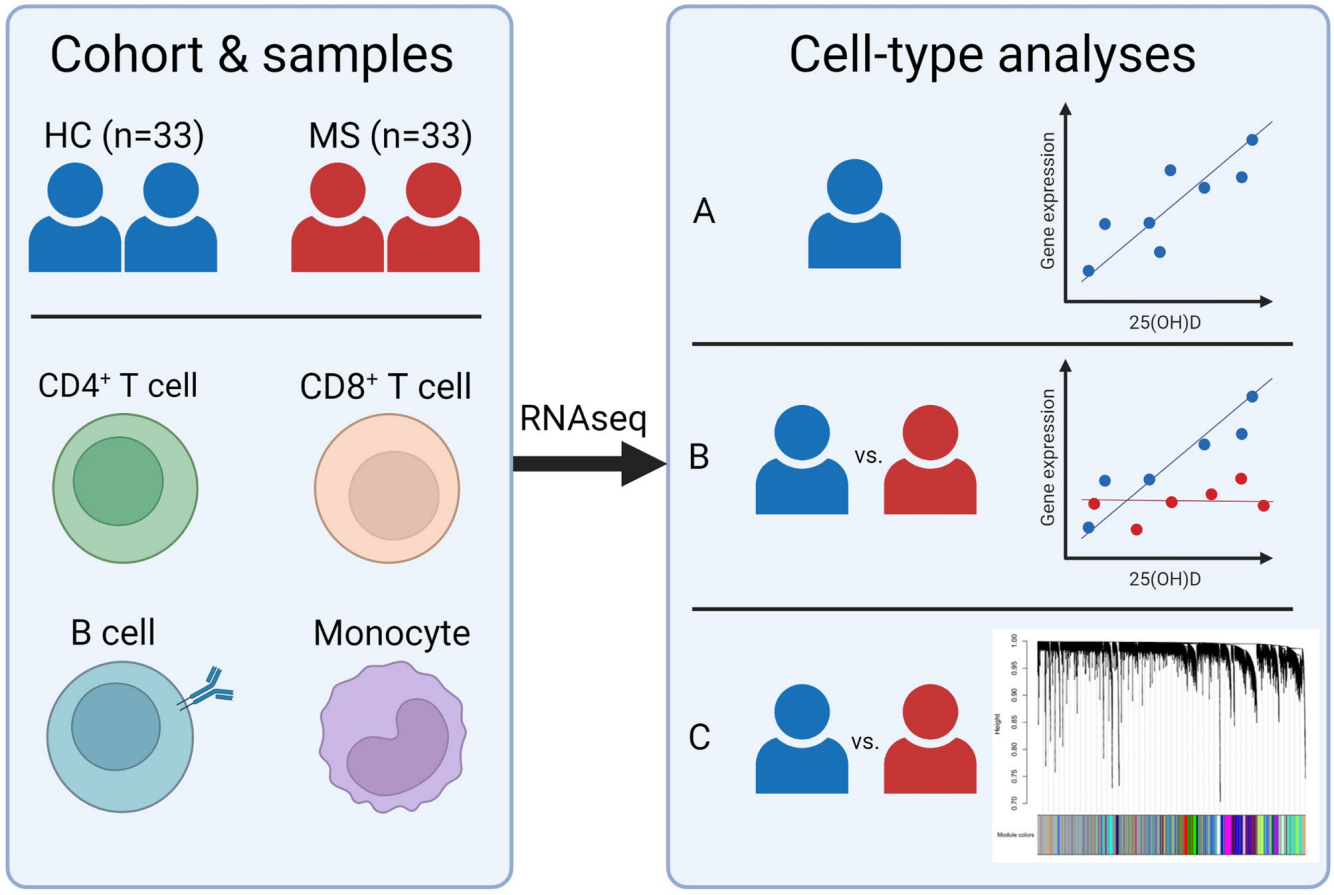
Overview of study design and analyses. We included 33 healthy controls and 33 participants with untreated multiple sclerosis. We collected blood by peripheral venepuncture and isolated immune cell subsets. RNA was isolated and sent for RNA sequencing. Transcriptomic analyses were performed within each cell type. (**A**) We first investigated in healthy controls for genes whose expression is correlated with plasma 25-hydroxyvitamin D (25(OH)D) level. (**B**) Next, we determined genes with differential association with endogenous vitamin D level between cases and controls. (**C**) We also utilized weighted gene co-expression network analysis (WGCNA) to identify modules of interconnected genes which are correlated with 25(OH)D in controls and cases. Figure created with BioRender.com.

We therefore investigated the immunobiology of vitamin D in MS cases and in non-MS controls through analysis of transcriptomic datasets generated by RNA sequencing from purified immune cell subsets. We first identified genes whose expressions correlated with endogenous 25(OH)D levels in healthy controls and explored functional significance of these regulated genes using both traditional bioinformatic pipelines and weighted gene co-expression network analysis (WGCNA). We next investigated whether genes are differentially regulated in association with endogenous vitamin D levels in people with MS compared to healthy controls, to provide insight into the mechanisms by which low vitamin D status may confer MS risk (Fig. 1).

## Results

We included 33 healthy participants and 33 participants with relapsing–remitting MS in this study. Included participants had both plasma and immune cell subset RNA collected at the same time. This allowed assessment of contemporaneous plasma 25(OH)D levels, and gene expression (Table 1). MS cases had not yet commenced on immunomodulatory treatment at the time of sample collection. Disability as measured on the ordinal Expanded Disability Status Scale (EDSS; range 0–10) of MS cases was mild (< 3) in most participants. Median 25(OH)D level was 70 nmol/L in the healthy control group and 90 nmol/L in the MS case group. Vitamin D deficiency at plasma 25(OH)D level < 30 nmol/L was present in one control participant and three MS cases.

**Table 1:** Participant characteristics.

	Healthy controls (n = 33)	Multiple sclerosis cases (n = 33)
Age, years, median (IQR)	32.4 (26.2–38.3)	38.1 (28.5–45.5)
Sex, n (%)		
Female	22 (66.7)	22 (66.7)
Male	11 (33.3)	11 (33.3)
25(OH)D level, nmol/L, median (IQR)	70 (61–86)	90 (58.0–106)
Time from first symptoms, years, median (IQR)	–	3.29 (0.76–8.75)
EDSS score, median (IQR)	–	1.5 (1–2)
Transcriptomic datasets included, n (%)		
CD4	33 (100)	29 (87.9)
CD8	33 (100)	27 (81.8)
B cell	30 (90.9)	26 (78.8)
Monocyte	29 (87.9)	29 (87.9)

*25(OH)D* 25-hydroxyvitamin D, *EDSS* Expanded Disability Status Scale.

### Genes and pathways modulated by vitamin D in healthy controls

We first performed correlation analysis between immune cell subset specific gene expression profiles and plasma 25(OH)D levels in non-MS controls. We then plotted *P*-value histograms of gene expression correlation significance levels with 25(OH)D levels. These histograms had a clear right-skewed distribution of *P*-values for CD4^+^ and CD8^+^ T cells, consistent with many gene transcripts correlated with endogenous vitamin D in healthy individuals (Supplementary Fig. 1).

**Figure 2:**
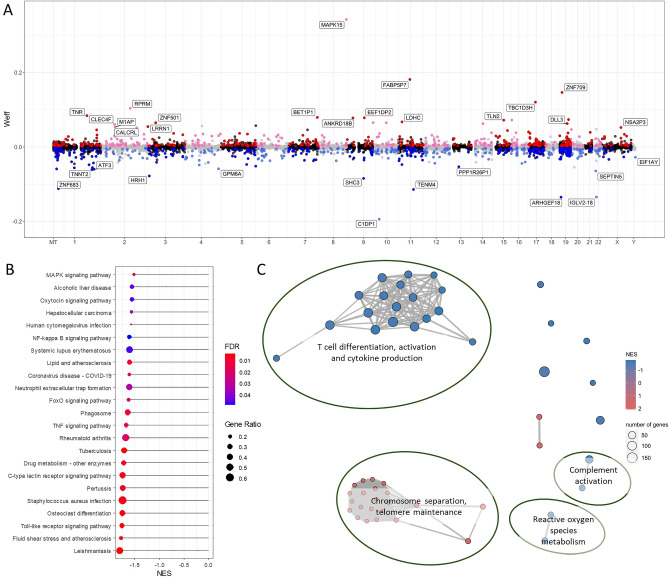
Genes and pathways correlated with vitamin D status in CD4^+^ T cells from healthy controls. (**A**) Mirror Manhattan plot of gene-vitamin D correlation analysis. Each dot represents a gene, x-axis represents gene location in the genome, y-axis represents weighted effect statistic (Weff) defined as − log_10_*P*-value * (log_2_ change in expression per 1 nmol/L change in 25(OH)D level). Red dots represent genes positively correlated with vitamin D level with *P* < 0.05, and blue dots represent genes negatively correlated and *P* < 0.05. (**B**) Gene set enrichment analysis (GSEA) using the KEGG Pathway database, shows negative enrichment of pathways involved in immune and cellular signaling pathways (FDR < 0.05). Gene ratio in GSEA is the ratio of core genes annotated in a term. (**C**) Enrichment map of GSEA results of Gene Ontology Biological Process terms. Each node represents an enriched gene set (FDR < 0.05), thickness of lines between node represent degree of overlap in genes between sets, and node color represents Normalized Enrichment Score (NES).

For CD4^+^ T cells, 1079 genes had expressions correlated with 25(OH)D level (unadjusted *P* < 0.05), of which 521 and 558 genes were positively and negatively correlated, respectively (Fig. 2A; Supplementary Table 1 includes results for significantly correlated genes of each cell type). No genes reached the threshold for statistical significance based on FDR < 0.05. To identify biological pathways modulated by vitamin D, we performed gene set enrichment analysis (GSEA). This analysis combines the signals from multiple genes to determine regulated pathways^15^ (Supplementary Table 2). In the KEGG Pathway database, we identified downregulation (as inferred from negative enrichment scores) of several immune-related pathways (Fig. 2B). These included signaling pathways involved in inflammation such as TNF-alpha and NF-κB signaling, autoimmune disease and infection pathways, and mitogen-activated protein kinase (MAPK) signaling pathways. The Hallmark gene set “TNF-alpha signaling via NF-κB” was enriched by genes negatively correlated with vitamin D (FDR < 0.0001). We found 52 significantly enriched Gene Ontology Biological Process terms which clustered into several key biological themes (Fig. 2C). Processes involved in chromosome separation and telomere maintenance were upregulated, whereas immune processes and p38 MAPK cascade were downregulated. Overall, these functional enrichment results strongly support an anti-inflammatory effect by vitamin D on CD4^+^ T cells.

**Figure 3:**
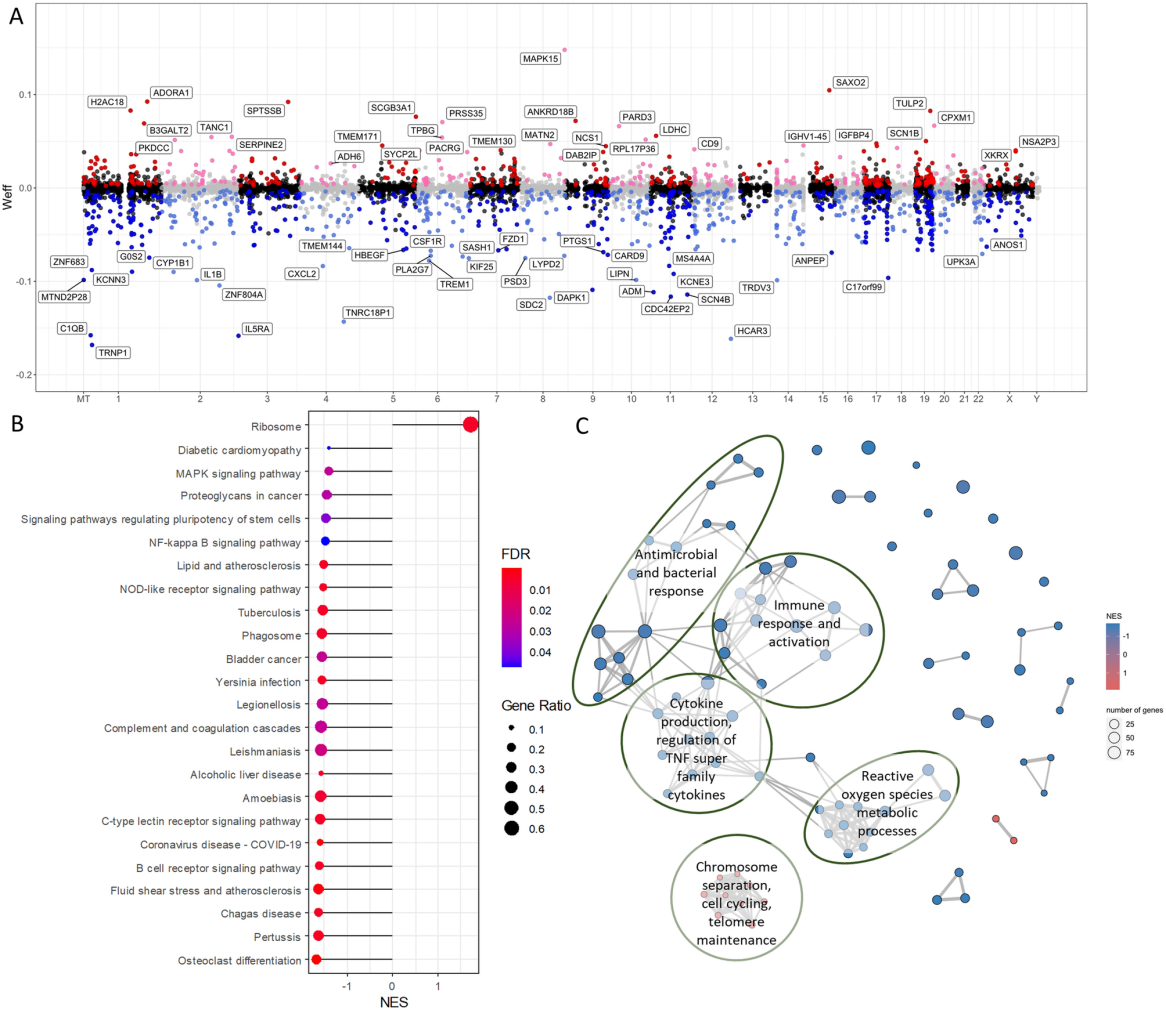
Genes and pathways correlated with vitamin D status in CD8^+^ T cells from healthy controls. (**A**) Mirror Manhattan plot of gene-vitamin D correlation analysis. Each dot represents a gene, x-axis represents gene location in the genome, y-axis represents weighted effect statistic (Weff) defined as − log_10_*P*-value * (log_2_ change in expression per 1 nmol/L change in 25(OH)D level). Red dots represent genes positively correlated with vitamin D level with *P* < 0.05, and blue dots represent genes negatively correlated and *P* < 0.05. (**B**) Gene set enrichment analysis (GSEA) using the KEGG Pathway database, shows negative enrichment of pathways involved in immune signaling and infectious disease pathways, and positive enrichment of the ribosome pathway (FDR < 0.05). Gene ratio in GSEA is the ratio of core genes annotated in a term. (**C**) Enrichment map of GSEA results of Gene Ontology Biological Process terms. Each node represents an enriched gene set (FDR < 0.05), thickness of lines between node represent degree of overlap in genes between sets, and node color represents Normalized Enrichment Score (NES).

In CD8^+^ T cells, we identified 1188 genes correlated with 25(OH)D level, 438 and 750 of which were positively and negatively correlated, respectively (Fig. 3A). Functional enrichment analysis (Supplementary Table 3) using the KEGG database identified positive enrichment in the ribosome pathway, and negative enrichment of remaining pathways involved in responses to infections and cellular signaling including NF-κB and MAPK signaling (Fig. 3B). The Hallmark “TNF-alpha signaling via NF-κB” gene set was negatively enriched (FDR = 0.001), concordant with the CD4^+^ T cell dataset. Enriched Gene Ontology terms revealed upregulation of chromosomal, cell cycling and telomere processes, and downregulation of immune responses and cytokine production (Fig. 3C). Similar to what we found in CD4^+^ T cells, these results support an immunoregulatory action of vitamin D on CD8^+^ T cells.

We further identified 858 and 731 genes whose expressions were significantly correlated with 25(OH)D levels in monocyte and B cells, respectively (*P* < 0.05; Supplementary Fig. 2). For monocytes, GSEA demonstrated negative enrichment of the Hallmark gene set “TNF-alpha signaling via NF-κB”, which was also enriched in CD4^+^ and CD8^+^ T cells, and positive enrichment of gene sets associated with RNA metabolism. There were no significantly enriched pathways identified in B cells.

### Regulation of MS risk genes by vitamin D

**Figure 4:**
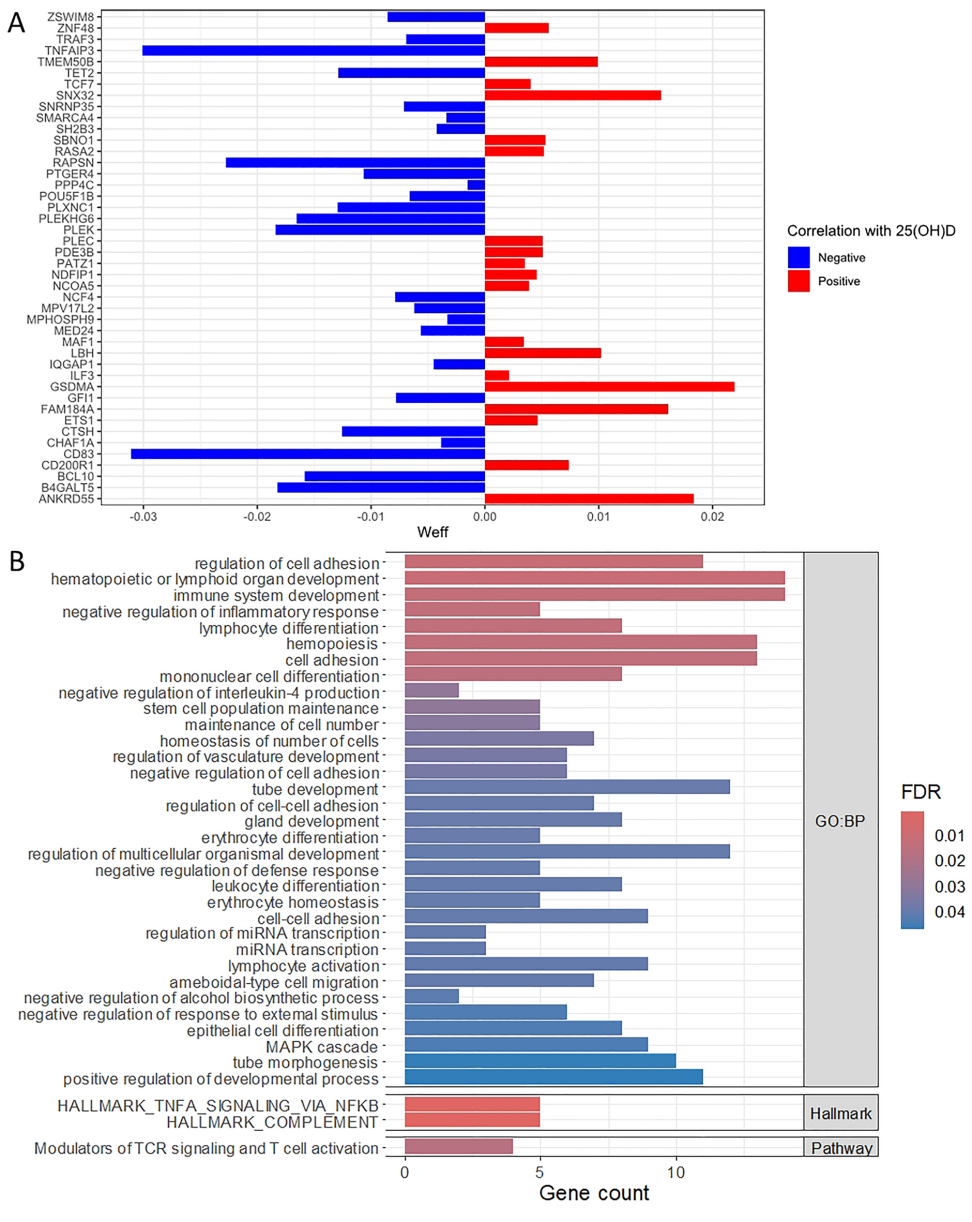
Vitamin D-modulated genes in CD4^+^ T cells from healthy controls which are multiple sclerosis susceptibility genes. (**A)** Plot of the 44 multiple sclerosis susceptibility genes whose expressions are significantly correlated with 25(OH)D level (*P* < 0.05). X-axis represents the weighted effect statistic (Weff) defined as − log_10_*P*-value * (log_2_ change in expression per 1 nmol/L change in 25(OH)D level). (**B**) Functional enrichment analysis of these genes shows involvement in immune cell differentiation, TNF-alpha signaling and miRNA transcriptional functions. X-axis represents the number of genes of interest present in the annotated term.

To determine if vitamin D might regulate the expression of MS susceptibility genes in immune cells, we tested for enrichment of MS risk genes, prioritized from susceptibility variants identified by a recent genome wide association study^16^. The vitamin D-correlated gene lists derived from our non-MS control transcriptomic datasets showed significant over-representation of MS risk gene expression in CD4^+^ T cells (*P* = 0.01) but not in other immune cell subsets. In CD4^+^ T cells, 44 genes were both MS susceptibility genes and regulated in association with vitamin D level. Of these, 25 genes (56.8%) were negatively correlated with 25(OH)D level (Fig. 4A). We conducted over-representation analysis of these 44 genes to delineate their functional significance. There were 33 Gene Ontology Biological Process terms significantly enriched (FDR < 0.05), with involvement in functions of immune cell differentiation, negative enrichment of inflammatory and defense responses, MAPK cascade and miRNA transcription (Fig. 4B). Three further terms were enriched, including TNF-alpha signaling. These findings support regulation of several MS susceptibility genes by vitamin D in CD4^+^ T cells, and modulation of T cell function and TNF-alpha signaling as potential mechanisms by which vitamin D influences MS risk.

### Differences in response to vitamin D between healthy controls and MS cases

We next investigated potential differential vitamin D-gene expression correlation between people with and without MS. We determined gene expression correlations with 25(OH)D level in MS cases and plotted *P*-value histograms for the results of each cell type (Supplementary Fig. 1). When compared to that of healthy controls, histogram distributions for the MS cohort were relatively flatter for CD4^+^ and CD8^+^ T cells.

**Figure 5:**
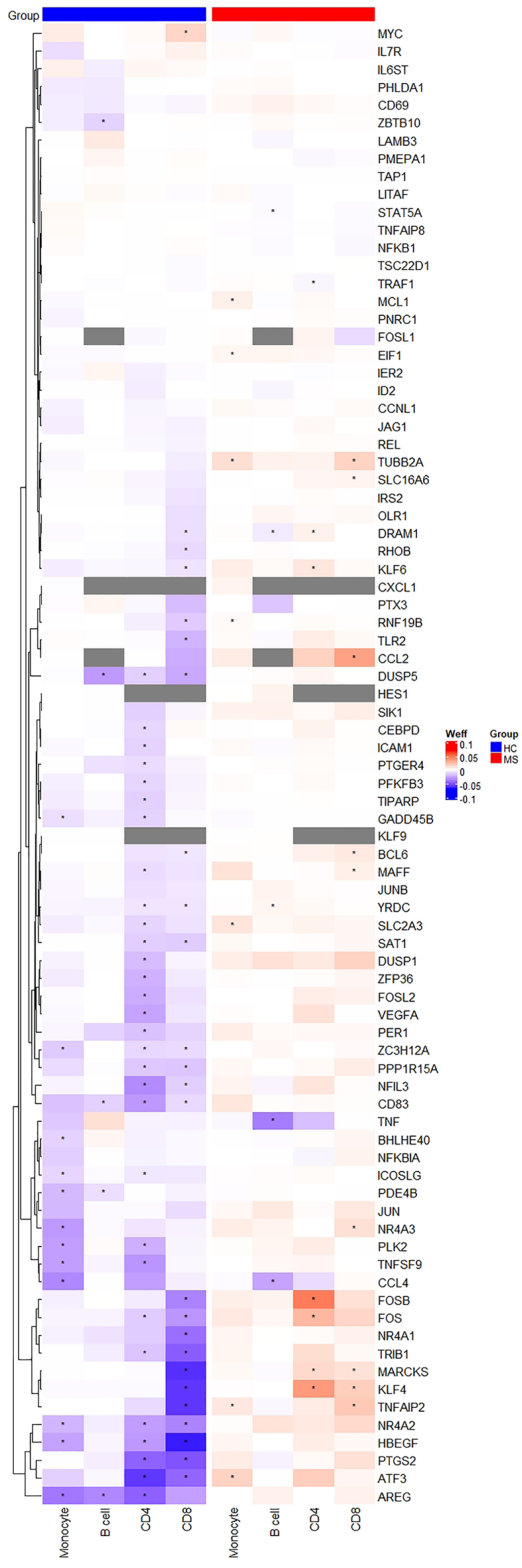
Heatmap of genes differentially correlated with endogenous vitamin D level between multiple sclerosis (MS) case and healthy control (HC) and leading to enrichment for TNF signaling. All genes which contributed to enrichment of Hallmark “TNF-alpha signaling via NF-κB” for any cell type were included in rows. Columns represent immune cell subsets, split by group (HC or MS). Tile color represents weighted effect statistic (Weff = − log_10_*P*-value * (log2 change in expression per 1 nmol/L change in 25(OH)D level). * Represents *P* < 0.05 for correlation between gene expression and 25(OH)D level. Grey tiles indicate genes not expressed in respective cell types.

We next identified genes with differential association with plasma 25(OH)D level between cases and controls and assessed if gene expression-25(OH)D level could predict MS case status (*P* < 0.05). We finally performed functional enrichment analysis on the resultant gene lists. We focused on the results of our functional enrichment analysis as this combines signals from multiple genes and therefore increased our ability to detect differential responses to vitamin D between MS cases and controls at the pathways level. We identified 912, 2029, 984 and 1144 genes from CD4^+^, CD8^+^, monocyte and B cell samples, respectively (*P*-value histograms in Supplementary Fig. 3; Supplementary Table 4 includes tabled results of differentially correlated genes by cell type). Functional analyses again revealed significant enrichment of TNF-alpha signaling in all cell types (Fig. 5; Supplementary Table 5 contains results of significantly enriched gene sets for each cell type). Many of these genes were negatively correlated with increasing 25(OH)D level in healthy controls, whereas this relationship was not seen in MS cases or for several genes was inverted. A number of other gene sets were enriched in CD8^+^ T cells. These included gene sets involved in RNA metabolism, protein localization, mitochondrial organization and gene expression, oxidative phosphorylation, and viral responses. Collectively, these findings strongly suggest that the association of gene expression and Vitamin D level in MS cases and controls is different.

### Vitamin D-regulated pathways identified through gene co-expression network analysis

Multiple gene co-expression patterns were tested using WGCNA, which is a method that utilizes gene correlation networks to identify modules of highly interconnected genes^17^. These modules can then be summarized by their respective module eigengenes (defined as the first principal component of each module’s expression matrix) and correlated with phenotypic data, with modules of interest interrogated further through functional enrichment analysis. Two networks can be compared through detection of modules common to both (in our study, healthy controls and MS cases), referred to as consensus modules^18^. We employed WGCNA, which summarizes our high-dimensional transcriptomic data to several modules before correlation with 25(OH)D level, to complement our traditional single-gene bioinformatic approach (which identified correlations for each gene) and to further investigate differences in vitamin D response between healthy controls and MS cases.

**Figure 6:**
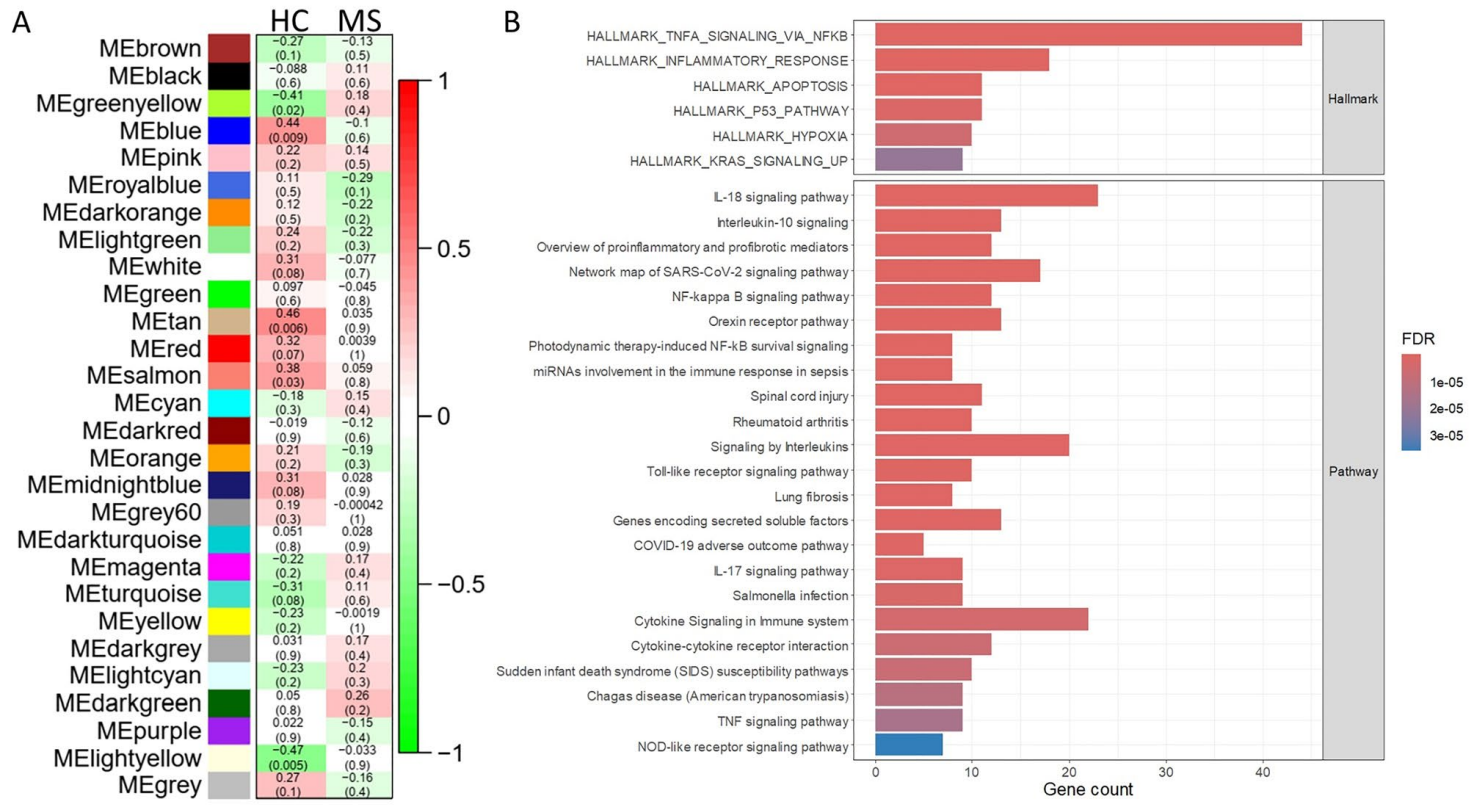
Vitamin D-regulated pathways in CD4^+^ T cells determined through weighted gene co-expression network analysis. (**A)** Consensus modules of healthy control (HC) and multiple sclerosis (MS) groups. For each module, the number represents the correlation coefficient between their respective module eigengene (ME) and plasma 25(OH)D level, and the number in brackets represents the *P*-value. (**B**) Functional enrichment results for the “light yellow” module, with the top 30 enriched Hallmark gene sets and pathways shown. X-axis represents the number of genes of interest present in the annotated term, and bar color represents the false discovery rate (FDR).

For CD4^+^ T cells, 27 consensus modules were detected (excluding the grey module to which non-clustered genes are assigned), five of which showed significant correlation with 25(OH)D levels in healthy controls (green yellow, light yellow, blue, tan, salmon) and none in MS cases (Fig. 6A). Genes in the “green yellow” module were enriched for RNA metabolic pathways, TNF-alpha signaling and IL2-STAT5 signaling (enrichment results for significant modules in Supplementary Table 6). The “light yellow” module was enriched for interleukin and cytokine signaling pathways such as TNF-alpha, IL-10 and IL-17, regulation of T helper (Th) cell differentiation, and vitamin D metabolism and response (including Gene Ontology Biological Process term “response to vitamin D”, FDR = 0.004; “Vitamin D in inflammatory diseases” pathway, FDR = 0.01) (Fig. 6B). The “blue” module showed enrichment for gene transcription and expression processes. We found no gene sets significantly enriched for the “tan” or “salmon” modules.

For CD8^+^ T cells, 25(OH)D level was significantly correlated with module eigengenes of 2/20 consensus modules for healthy controls (tan, blue) and one for MS cases (purple) (Supplementary Fig. 4). Interleukin-10 and cytokine signaling and regulation of T cell differentiation and activation were enriched for the “tan” module, and RNA metabolism and translation were enriched for the “blue” module (enrichment results in Supplementary Table 7). For the “purple” module, there was enrichment for leukocyte differentiation and activation, regulation of TNF and interleukin production, toll-like receptor signaling, other cellular signaling pathways including NF-κB and MAPK, and the vitamin D receptor pathway.

There were two significant modules for B cells, namely “light green” in healthy controls and “orange” in MS cases (Supplementary Fig. 5). Functional enrichment analysis identified TNF-alpha signaling as enriched in the “light green” module. No gene sets were enriched in the “orange” module. There were no modules associated with vitamin D level for monocytes (Supplementary Fig. 6).

In summary, results of WGCNA identify immune and metabolic processes as modulated by vitamin D, particularly in CD4^+^ and CD8^+^ T cells, and a greater number of modules associated with vitamin D levels in healthy controls than MS cases. These support a differential and reduced response in those with MS.

### Expression of vitamin D metabolism and receptor genes by case status

We investigated whether our observed differences in vitamin D responsiveness between cases and controls could be due to differential expression of genes involved in vitamin D metabolism and response. However, our analysis did not identify any evidence of differential expression of vitamin D metabolism or response genes between MS cases and non-MS controls (Supplementary Table 8).

## Discussion

**Figure 7:**
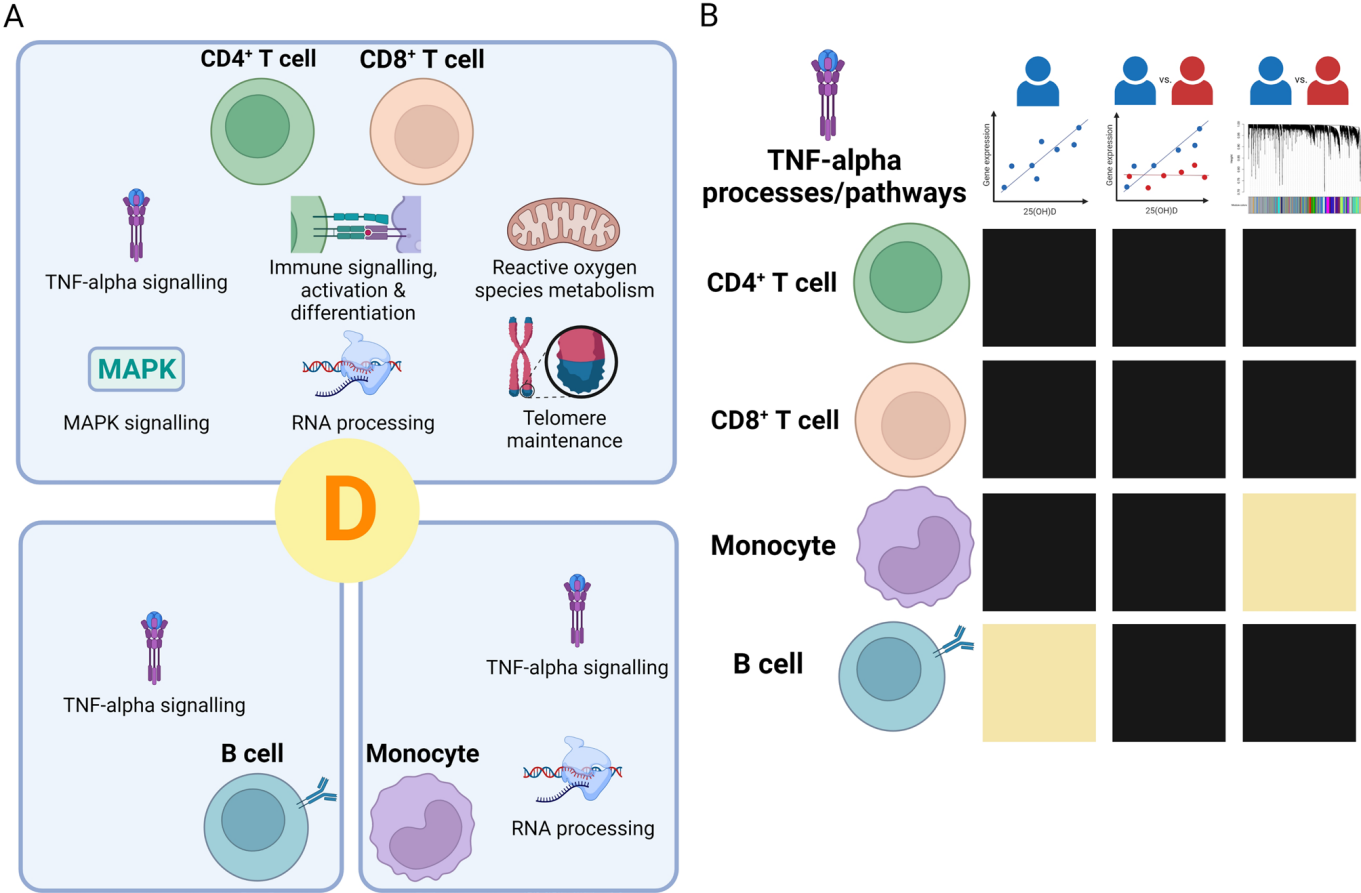
Summary of functional enrichment analyses results for vitamin D-correlated genes. (**A**) Significantly enriched pathways and processes within each immune cell type showed overlap across CD4^+^ and CD8^+^ T cells, including immune activation and differentiation and telomere maintenance. RNA metabolism and processing gene sets were enriched in T cells and monocytes, while TNF-alpha signaling was enriched across all four immune cell types. (**B**) Heatmap of enrichment for TNF-alpha signaling and production processes/pathways for each cell type (rows). Each column represents our primary analyses: gene correlation with plasma 25(OH)D in non-multiple sclerosis control samples (column 1), genes differentially correlated with 25(OH)D between cases and controls (column 2), and weighted gene co-expression network analysis (WGCNA) (column 3). Black represents significant enrichment (FDR < 0.05), while beige indicates absence of enrichment. Figure created with BioRender.com.

We successfully identified pathways associated with plasma 25(OH)D level in immune cell subsets (Fig. 7). Our study of healthy controls indicates that genes associated with immune processes, RNA metabolism and cellular signaling pathways are correlated with 25(OH)D in CD4^+^ and CD8^+^ T cells. Vitamin D-responsive genes in CD4^+^ T cells were enriched for MS risk gene loci. TNF-alpha signaling was differentially regulated between those with and without MS. Consensus analysis using WGCNA identified fewer vitamin D-associated modules in MS than non-MS controls, in support of a potential reduced response to vitamin D in cases.

We identified TNF-alpha signaling pathways as associated with plasma vitamin D level across multiple immune cell types and, intriguingly, demonstrated differential association with vitamin D between those with and without MS. Some TNF-alpha pathway-associated genes are in known MS risk loci, and, interestingly, several were associated with vitamin D in CD4^+^ T cells from non-MS controls. TNF-alpha is a cytokine with a wide spectrum of functions, including both pro-inflammatory and immunoregulatory roles via its receptors TNFR1 and TNFR2, respectively. It can be produced by innate and adaptive immune cells, as well as non-immune cell types such as glial cells^19–22^. The role of TNF-alpha in MS is complex. Elevated TNF-alpha levels detected either peripherally or in cerebrospinal fluid of people with MS are associated with disease activity^23–25^. However, the use of TNF inhibitors to treat MS instead led to disease aggravation^26^. The non-selective inhibition of both TNFR1 and TNFR2 signaling could explain this, as TNFR2 promotes anti-inflammatory Treg function as well as remyelination, whereas TNFR1 signaling is thought to be more pro-inflammatory^27^.

Our transcriptomic analyses support a role for vitamin D in the regulation of TNF-alpha signaling, including downstream pathways (NF-κB and MAPK), and a likely role in MS pathogenesis mediated via CD4^+^ T cells. Prior evidence has shown that addition of vitamin D reduces TNF-alpha production in immune cells in vitro^28,29^. Furthermore, serum TNF-alpha levels are known to be negatively correlated with serum 25(OH)D in healthy women^30^. Based on our results and those of previous studies, it is likely that vitamin D regulates both downstream signaling of TNF-alpha and TNF-alpha levels to promote an anti-inflammatory phenotype. One other possibility, in the context of MS, is that vitamin D may skew TNF-alpha signaling through TNFR2 rather than TNFR1. A study of smooth muscle cells showed that vitamin D could induce shedding of TNFR1, but not TNFR2, from the cell surface and therefore reduce signaling via TNFR1^31^. However, it is not known if this also occurs in immune cells and is an area open to future study. Overall, our results suggest that dysregulated TNF-alpha signaling either in the setting of vitamin D deficiency or vitamin D hyporesponsiveness, could be important for determining MS pathogenesis. This could also be a mechanism by which vitamin D might promote immune tolerance and prevent autoimmunity.

Vitamin D deficiency has been epidemiologically linked with elevated risk of autoimmune diseases including MS, rheumatoid arthritis, inflammatory bowel disease, systemic lupus erythematosus and inflammatory skin diseases^10,32–35^. However, the pathophysiology of these diseases and links to vitamin D deficiency are not understood. In a recent randomized placebo-controlled trial of vitamin D supplementation in adults over 50 years, investigators found a 22% reduction in risk of incident autoimmune disease over a median 5.3 years follow-up in those randomized to vitamin D3^36^. Much of our understanding of the immunobiology of vitamin D has been derived from animal and in vitro studies, the latter using cells cultured with calcitriol or analogues at supraphysiological concentrations. In these models, investigators can define functional and transcriptional regulation in both innate and adaptive immune cells^5,6,37^. Importantly, our study adds to this understanding by correlating vitamin D levels and gene expression profiles in immune cell subsets from both healthy people and people with untreated MS.

Our results support CD4^+^ and CD8^+^ T cells as vitamin D-responsive in a physiological setting, with both cellular metabolic and immunomodulatory effects. We identified gene enrichment in cell cycling and chromosomal separation pathways, concordant with the recognized role of vitamin D signaling in the priming and proliferation of naïve T cells^38^. We also identified enrichment of telomere maintenance processes, supporting previous findings that higher vitamin D levels are associated with longer leukocyte telomere length^39^. Our GSEA results also demonstrated downregulation of MAPK signaling, including the p38 MAPK cascade. Interestingly the p38 MAPK is involved in the alternative T cell receptor signaling pathway and has been shown to upregulate VDR expression and vitamin D responsiveness in activated T cells^38^. Inhibition of p38 MAPK signaling via the T cell receptor in CD4^+^ T cells led to reduced production of IFN-γ and IL-17 and reduced severity of animal autoimmune disease models for MS and rheumatoid arthritis^40^. Several studies have shown that vitamin D can promote anti-inflammatory effects via inhibition of p38 MAPK activation^41–43^. Considered together, these findings inform vitamin D’s immune homeostatic role in T cells and also propose possible mechanisms by which vitamin D deficiency can contribute to autoimmunity.

Multiple immune signaling pathways were enriched by vitamin D-correlated genes of T cells from healthy controls. These included IL-2, IL-10 and IL-17 signaling pathways. In terms of genomic regulation of cytokine expression, vitamin D is known to repress *IL2* and *IL17A* transcription through VDR binding at their promoter regions and competition for DNA binding with the transcription factor NFAT^44–47^. Vitamin D also induces Foxp3 in CD4^+^ T cells to negatively regulate IL-17A production^44^. Cell culture with calcitriol demonstrated decreased production of IL-2 and IL-17^7,44,48–50^, and increased IL-10 production with promotion of Treg differentiation^7,49,51,52^. Furthermore, a recent study delineated a complement-induced autocrine/paracrine vitamin D autoregulatory loop in CD4^+^ T cells which represses Th1 and Th17 responses and activates a Treg program^52^. Considered together, these pieces of evidence support an important immune homeostatic role for vitamin D. Plausibly, dysregulation of this system, for instance in the setting of vitamin D deficiency, could lead to inappropriately sustained inflammation and loss of tolerance towards self-antigens.

In our consensus analysis using WGCNA, we detected more vitamin D-correlated modules in non-MS controls than MS cases. This suggests immune cells from people without MS are more vitamin D responsive. Prior evidence of a “blunted” vitamin D response in those with MS are smaller changes in serum 25(OH)D level and attenuated alterations in plasma metabolites following vitamin D supplementation in those with MS compared to those without^53,54^. Culture with calcitriol of peripheral blood mononuclear cells isolated from MS cases showed smaller decreases in TNF-alpha production compared to cells from healthy controls, again suggesting a reduced responsiveness of the MS group^55^. Enzymes involved in vitamin D metabolism are important regulators of vitamin D levels, and are also MS susceptibility genes, in particular CYP27B1, CYP24A1 and CYP2R1^16^. Here we did not assess the genotypes of the study participants at vitamin D-related risk loci. However, nor did we identify any differences in the expression of vitamin D metabolism genes by case–control status. Thus, it appears unlikely that the observed differences in vitamin D-associated gene expression between MS cases and controls are associated with changes in vitamin D metabolism pathways. Other potential explanations for these case vs control differences in vitamin D immune gene regulation could include altered isoform proportions of genes involved in vitamin D metabolism and response^56^ and epigenetic differences, both inherited and acquired^57^. Further work is, however, required to confirm and elucidate mechanisms for a differential response to vitamin D in those with, and at risk of, MS. A recent study which investigated the effect of high dose vitamin D3 supplementation in participants with MS identified changes in immune subset gene expression after 6 months^58^. Future research of the effects of vitamin D3 supplementation on the immune cell transcriptome, particularly among individuals with vitamin D deficiency and comparisons between those with and without MS, will yield additional insights into the immunoregulatory effects of vitamin D and how vitamin D response differs in people with and without MS.

Our study of transcriptomic changes by vitamin D using human immune cells has a number of strengths. We isolated immune cell subsets which allowed us to determine cell-specific signals, in contrast to studies which utilize whole blood or peripheral blood mononuclear cells. We utilized GSEA for functional analyses of our healthy control datasets, which combines signals from multiple genes and increases power to detect relevant pathways. We also applied a gene co-expression network approach to complement our generalized linear modelling with edgeR. Enrichment for vitamin D-related pathways provided additional validation of our results. There are however, several limitations. Our cross-sectional design and sample size limit our power, particularly at the single gene level in which we did not identify any FDR significant genes correlated with vitamin D level. We therefore combined the signals from multiple genes to maximise our power to detect associations with vitamin D level through our analytical pipelines which utilize functional enrichment analysis and WGCNA. Due to our limited sample size, we were unable to perform subgroup analysis based on 25(OH)D level stratification. Participants in this study were originally recruited for a previous genomics study and vitamin D supplementation data was not collected. Therefore, we were unable to investigate any specific gene expression changes due to vitamin D supplementation. Our correlational approach to gene expression and vitamin D level means we can only infer on vitamin D immunoregulation, although our results are supported by in vitro and animal-based studies of vitamin D; we are planning human vitamin D supplementation studies which will be better placed to determine vitamin D-induced immune effects. We examined peripherally-collected immune cells which are most accessible but may not be most relevant in regards to investigating pathophysiologic alterations in MS.

Overall, we analyzed purified human immune cell subset transcriptomes and identified numerous CD4^+^ and CD8^+^ T cell gene expression profiles as correlated with endogenous vitamin D levels. This extended to immune cell pathways of TNF-alpha and MAPK signaling, and chromosome and telomere maintenance processes. Vitamin D-associated genes in CD4^+^ T cells were enriched for MS risk genes, which supports the importance of this cell type in MS pathogenesis. We detected differential response to vitamin D in TNF-alpha signaling between those with and without MS, and supportive evidence for an overall reduced vitamin D responsiveness in people with MS. A dysregulated vitamin D response and consequent impaired immunoregulatory effect could, in part, explain vitamin D’s role in MS risk and pathogenesis. Future studies of vitamin D supplementation and utilization of multiple omics technologies will help further elucidate the in vivo effects of vitamin D in physiologic and pathophysiologic contexts, and these insights will likely allow us to delineate strategies to prevent and better treat MS and other autoimmune diseases.

## Methods

### Study population

Participants with relapsing–remitting MS were recruited from Box Hill Hospital and Royal Melbourne Hospital, Victoria, Australia, between 2014 to 2016. MS cases were diagnosed by a neurologist and met McDonald’s diagnostic criteria^59^. Cases were not on immunomodulatory treatment at time of blood sampling. Healthy controls who did not have any medical history of neurological or autoimmune disease were recruited across the same time period. Cases and controls were both of European ethnicity to avoid confounding due to ethnicity and as most people with MS in Australia are of European ethnicity.

### Ethics approval and consent to participate

The study was conducted according to the Declaration of Helsinki principles and approved by the Human Research Ethics Committees of Royal Melbourne Hospital and Box Hill Hospital, Victoria, Australia. Written informed consent was obtained from all participants.

### Plasma vitamin D level assessment

Blood was collected by peripheral venepuncture, plasma isolated and then frozen at − 80 °C on the same day as collection. Plasma samples were sent to Melbourne Pathology and 25(OH)D levels quantified by the LIAISON 25 OH Vitamin D TOTAL Assay, which assesses both 25(OH)D_2_ and 25(OH)D_3_.

### Peripheral immune cell collection and isolation

Up to 110 mL of venous blood was collected in EDTA vacutainer tubes (BD). To isolate peripheral blood mononuclear cells (PBMC), up to 70 mL of blood was diluted with PBS at a 1:2 ratio and then gently overlaid over 15 mL Histopaque-1077 (Sigma-Aldrich) in 50 mL tubes. Tubes were centrifuged at 400*g* with brakes off for 30 min at room temperature. The PBMC-enriched layer was collected, washed twice with PBS and cells counted. Collected cells were incubated with human CD19 and CD8 microbeads, and then B cells and CD8^+^ T cells, respectively, were positively selected using LS columns as per manufacturer’s instructions (Miltenyi Biotec). The negative fraction after CD8^+^ T cell selection was used to isolate CD4^+^ T cells using CD4 microbeads (Miltenyi Biotec) as per manufacturer’s instructions. For monocyte isolation, up to 40 mL of blood was centrifuged at 400*g* with brakes off for 15 min at room temperature. Buffy coat was collected and incubated with RosetteSep human monocyte enrichment cocktail (Stemcell Technologies). This was layered over 5 mL Histopaque in 15 mL tubes and centrifuged at 1200*g* with brakes off. The enriched cell layer was collected, washed, counted and labelled with human CD14 microbeads for positive selection by autoMACS Pro separator (Miltenyi Biotec). Purity of isolated immune cell subsets were assessed by standard flow cytometry protocols on a Cyan Flow cytometer (Beckman Coulter). Samples with purity greater than 90% were included.

### RNA isolation and sequencing

RNA was isolated from immune subsets using the Qiagen RNeasy Mini Kit as per manufacturer’s protocol with DNase digestion. RNA yield and quality were assessed by NanoDropND-1000 or the Qiagen QIAxpert System. RNA samples were sent to the Australian Genome Research Facility for library preparation and RNA sequencing. For B cell and monocyte samples, cDNA libraries were generated with the TruSeq Stranded mRNA kit (Illumina) and 100 base pair single end reads produced on the Illumina HiSeq 2500 System, with approximately 40 million reads per sample generated. For CD4^+^ and CD8^+^ T cell samples, library preparation was with the Illumina Stranded mRNA kit and sequencing on the Illumina Novaseq 6000 system to generate approximately 30–40 million paired end reads per sample of 150 base pair length. Read quality was assessed with FastQC^60^ and reads mapped to the reference transcriptome by Kallisto^61^.

### Gene expression data pre-processing, normalization and correction of unwanted variation

Downstream processing and analyses were performed in R version 4.1.1^62^. Tximport was used to summarize transcript abundances to the gene level, scaled for average transcript length and library size^63^. Gene-level counts were imported by the package edgeR^64^. Principal components analysis was performed, and Principal Component 1 and Principal Component 2 were plotted to identify outlier samples which were then excluded. Genes with a minimum count of 10 across the number of samples in the smallest group were kept by use of the edgeR function “filterByExpr”. Count data were normalized using the trimmed mean of M-values method. We used RUVseq to estimate latent variables which represent unwanted technical variation^65^. The RUVg method was used which uses a list of housekeeping genes as negative controls^66^. This list of housekeeping genes was excluded for MS risk genes^16^ and any genes potentially modulated by vitamin D, as identified by previous in vitro and supplementation studies^67–69^, to avoid unwanted adjustment out of our signals of interest. The number of latent variables to include was assessed by visualizing adjusted principal component analysis and relative log expression plots^70^, as recommended by authors of RUVseq. Gene annotations were accessed through Ensembl (version 106) through the package biomaRt^71,72^.

### Gene expression analysis with plasma vitamin D level

edgeR was used to perform generalized linear modelling with the use of the quasi-likelihood framework^73^. Genes whose expressions were correlated with plasma 25(OH)D level (nmol/L) were determined, and models were adjusted for participant age and sex, as well as latent variables if required. We considered genes significant based on an unadjusted *P* < 0.05. As we expected limitations in power to detect correlations with 25(OH)D at the single gene level, we used our defined *P*-value cut-off to facilitate downstream functional enrichment analyses which combines the signals from multiple genes to better detect vitamin D associations at the pathway/processes level.

### Differential expression analysis of vitamin D metabolism genes

Genes involved in vitamin D metabolism were retrieved from the WikiPathways “Vitamin D metabolism (Homo sapiens)” pathway^74^. edgeR was used to assess for differential expression of these genes between cases and controls, with the model adjusted for age, sex and 25(OH)D level. Significance was defined by *P* < 0.05.

### Over-representation analysis for MS risk genes

Non-MHC MS candidate risk genes were determined by a recent genome-wide association study^16^. We assessed for enrichment of these risk genes in our lists of vitamin D-correlated genes identified for each immune subset of healthy controls through one-sided Fisher’s exact test. A significant enrichment was defined by *P* < 0.05.

### Modelling for genes differentially regulated by vitamin D between cases and controls

We used logistic regression to identify genes whose interaction with plasma 25(OH)D level predicted case–control status after adjustment for age, sex, gene expression and plasma 25(OH)D level for each immune subset (*P* < 0.05). These genes exhibit differential correlation of their expressions with vitamin D level between MS cases and healthy controls.

### Weighted gene co-expression network analysis (WGCNA)

WGCNA is a method which utilizes gene co-expression relationships to construct gene networks^17^. Modules of highly co-expressed genes are identified and summarized by their module eigengene. Module eigengenes can then be correlated with traits of interest (vitamin D level in our study) to determine modules of interest, following which the genes belonging to these modules can be used to perform functional enrichment analysis to determine relevant biological pathways. MS cases and healthy controls were compared by detection of consensus modules^18^. We used the WGCNA package to perform this analysis by each immune subset, and followed Tutorial II for consensus analysis as provided by the package’s authors (https://horvath.genetics.ucla.edu/html/CoexpressionNetwork/Rpackages/WGCNA/Tutorials/, accessed on 20th November 2021)^75^. Expression data adjusted for latent variables, if required, were used as input data. For network construction and consensus module detection, we chose the lowest soft threshold power which achieved a scale free topology model fit of at least 0.8. Eigengene networks for all cell types showed overall high preservation between controls and cases, as represented by high densities of their preservation networks (range 0.85–0.88). Module eigengenes were correlated with plasma 25(OH)D level to determine modules significantly correlated with vitamin D (*P* < 0.05) for cases and controls, respectively.

### Functional enrichment analysis and visualizations

For functional analysis of our healthy control samples, we employed gene set enrichment analysis (GSEA) which uses the ranked full gene list^15^. Genes were ranked in descending order of weighted effect statistic (Weff), defined as − log_10_(*P*-value) * (log_2_ change in expression per 1 nmol/L change in 25(OH)D level). For each gene set, GSEA calculates a normalized enrichment score which represents the amount of genes over-represented either among positively correlated genes (positive enrichment score) or negatively correlated genes (negative score). Significantly enriched gene sets were defined by a false discovery rate (FDR) < 0.05, as controlled by the Benjamini–Hochberg method^76^. Gene sets in the Molecular Signatures Database (Hallmark gene sets, canonical pathways and Gene Ontology sets) and KEGG Pathway database were assessed for enrichment, respectively^77,78^. GSEA and result visualization by enrichment map were performed by the package clusterProfiler, with other visualizations created by ggplot2^79,80^.

Enrichment analysis by over-representation analysis was performed in the online tool ToppGene using Hallmark, Gene Ontology and Pathway gene sets^81^. Benjamini–Hochberg-adjusted FDR < 0.05 was considered to be significant enrichment. Heatmap was generated by the ComplexHeatmap package^82^.

### Supplementary Information

Supplementary Information.

## Data Availability

The RNA sequencing datasets generated during and/or analysed during the current study are available in the European Genome-Phenome Archive under study EGA ID EGAS00001007254.
