# Supplementary material for: Transcriptomics identifies blunted immunomodulatory effects of vitamin D in people with multiple sclerosis
Source: Sci Rep. 2024 Jan 16;14:1436. doi: 10.1038/s41598-024-51779-0 (PMC10792011; doi:10.1038/s41598-024-51779-0)
Supplement: Supplementary file 1 — Supplementary Information. [file 41598_2024_51779_MOESM1_ESM.zip › Supplementary File 1 - Supplemental Information.pdf]

Supplemental Information for: Transcriptomics identifies blunted immunomodulatory effects of vitamin D in people with multiple sclerosis

Wei Z. Yeh,[1,2] Rodney Lea,[3,4], Jim Stankovich,[1], Sandeep Sampangi,[1,2] Louise Laverick,[5], Anneke Van der Walt,[1,2] Vilija Jokubaitis,[1,2] Melissa Gresle,[1,2,5, †] and Helmut Butzkueven[1,2,†]

[†]**These authors contributed equally to this work.**

Author affiliations:

1 Department of Neuroscience, Central Clinical School, Monash University, Melbourne, Victoria, Australia

2 Department of Neurology, Alfred Health, Melbourne, Victoria, Australia

3 School of Biomedical Sciences and Pharmacy, University of Newcastle, Newcastle, Australia

4 Centre for Genomics and Personalised Health, School of Biomedical Sciences, Queensland University of Technology, Brisbane, Australia

5 Department of Medicine, University of Melbourne, Melbourne, Victoria, Australia

Contents

Supplementary Fig. 1: P-value histograms for gene expression correlation with vitamin D level in healthy controls (HC) and multiple sclerosis (MS) groups by cell type 3

Supplementary Fig. 2: Mirror Manhattan plots of gene-vitamin D correlation analyses in healthy controls. 4

Supplementary Fig. 3: P-value histograms for genes whose interaction with vitamin D level predicted MS case-control status by cell type 5

Supplementary Fig. 4: Consensus modules and correlation with vitamin D level for CD8^{+} T cells 6

Supplementary Fig. 5: Consensus modules and correlation with vitamin D level for B cells 7

Supplementary Fig. 6: Consensus modules and correlation with vitamin D level for monocytes 8

Supplementary Table 8: Differential expression analyses between MS cases and healthy controls of genes involved in vitamin D metabolism 9

Supplementary table legends for supplementary table files 10

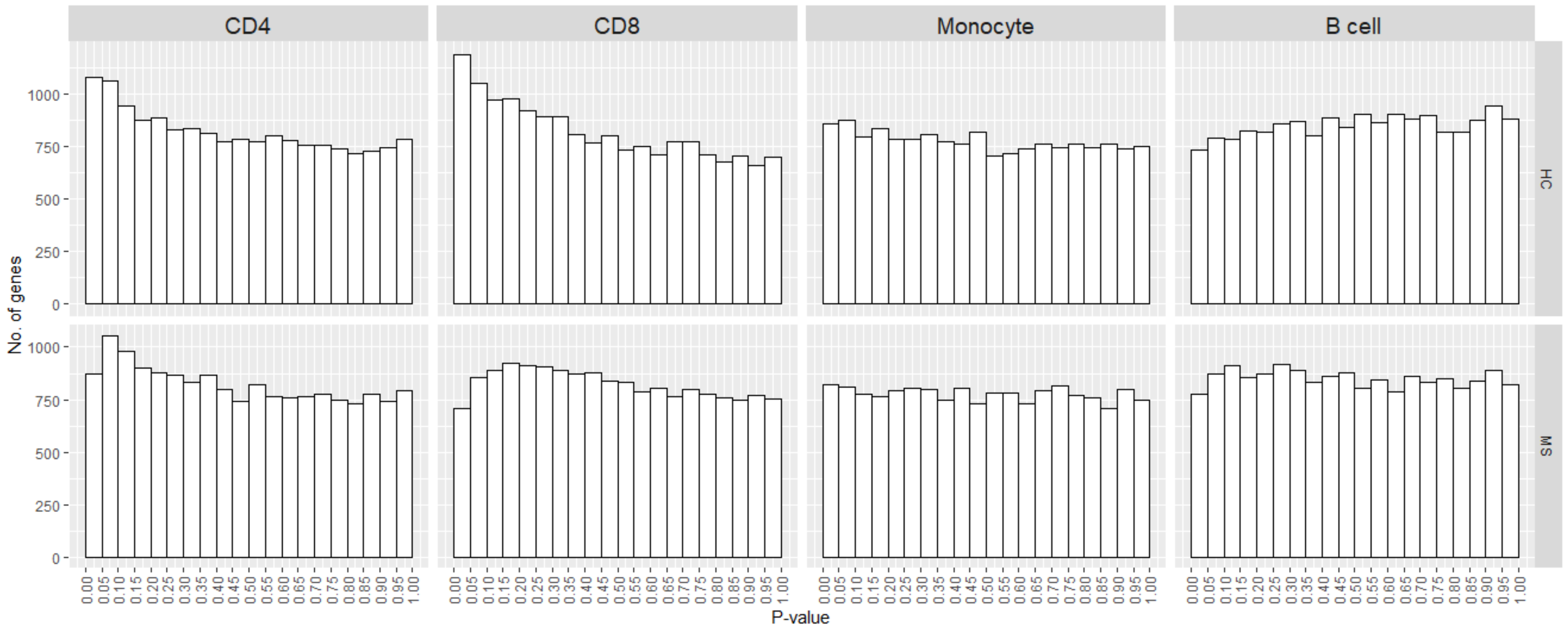

Supplementary Fig. 1: P-value histograms for gene expression correlation with vitamin D level in healthy controls (HC) and multiple sclerosis (MS) groups by cell type

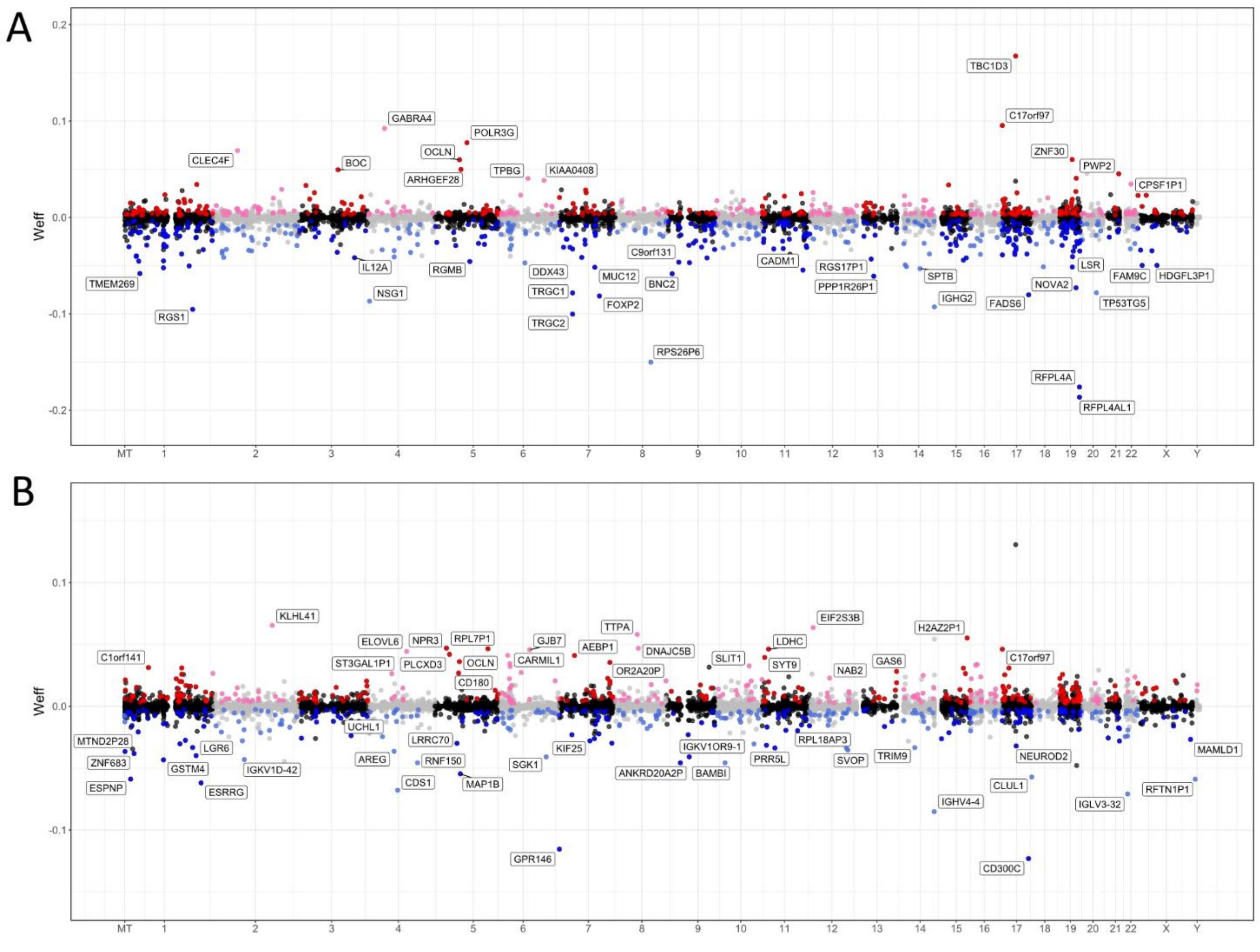

Supplementary Fig. 2: Mirror Manhattan plots of gene-vitamin D correlation analyses in healthy controls.

Plots depict analyses for monocyte (a) and B (b) cell types, respectively. Each dot represents a gene, x-axis represents gene location in the genome, y-axis represents weighted effect statistic (Weff) defined as -log10(P-value)*(log_2 change in expression per 1 nmol/L change in 25(OH)D level). Red dots represent genes positively correlated with vitamin D level with P<0.05, and blue dots represent genes negatively correlated and P<0.05.

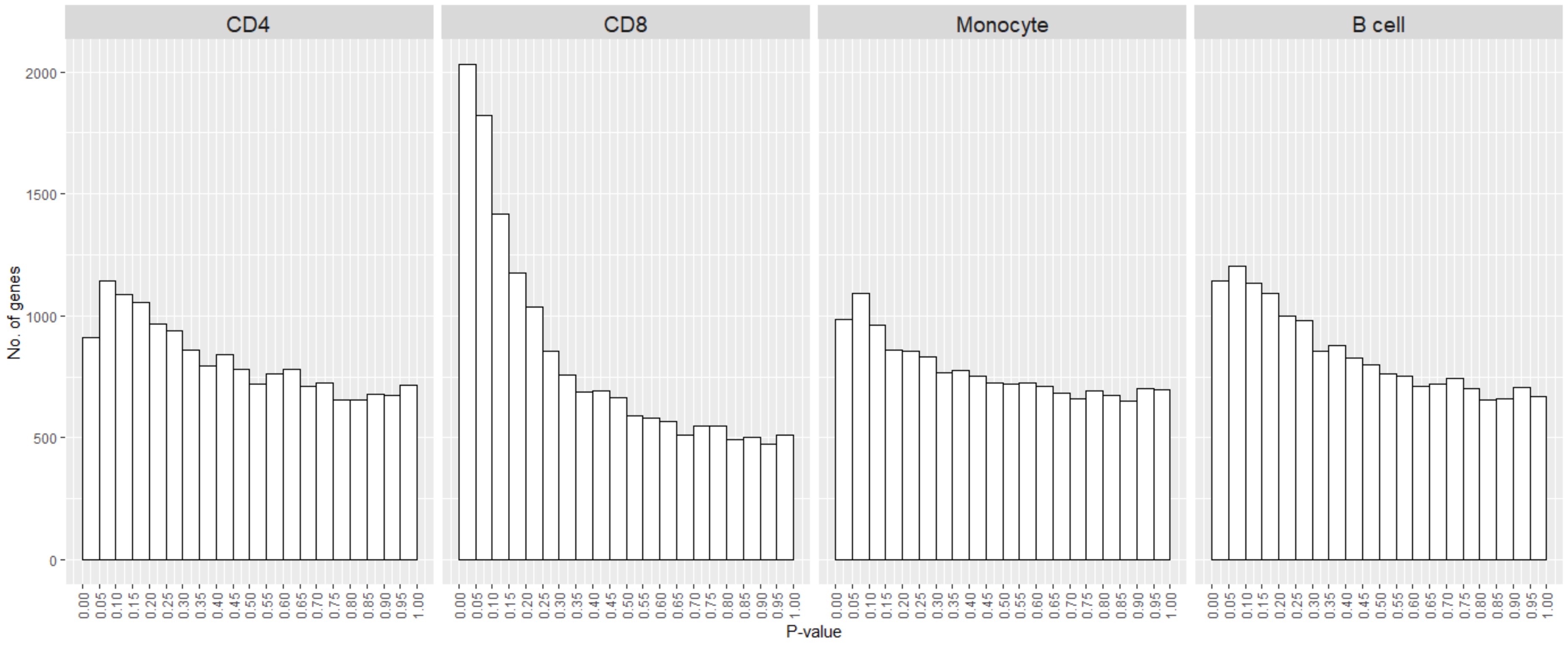

Supplementary Fig. 3: P-value histograms for genes whose interaction with vitamin D level predicted MS case-control status by cell type

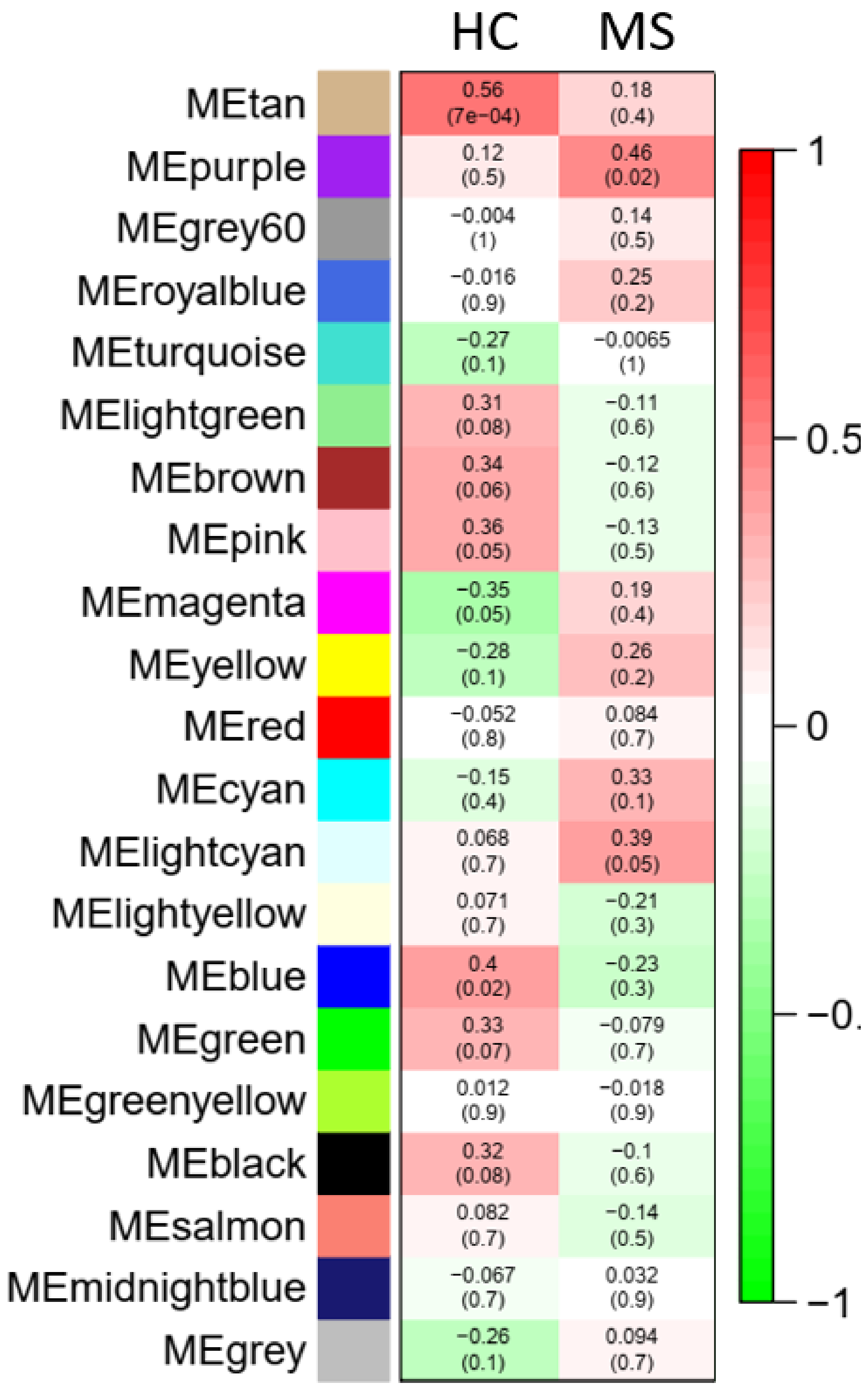

Supplementary Fig. 4: Consensus modules and correlation with vitamin D level for CD8⁺ T cells

Consensus modules of healthy control (HC) and multiple sclerosis (MS) groups were identified. For each module, the number represents the correlation coefficient between their respective module eigengene (ME) and plasma 25(OH)D level, and the number in brackets represents the p-value.

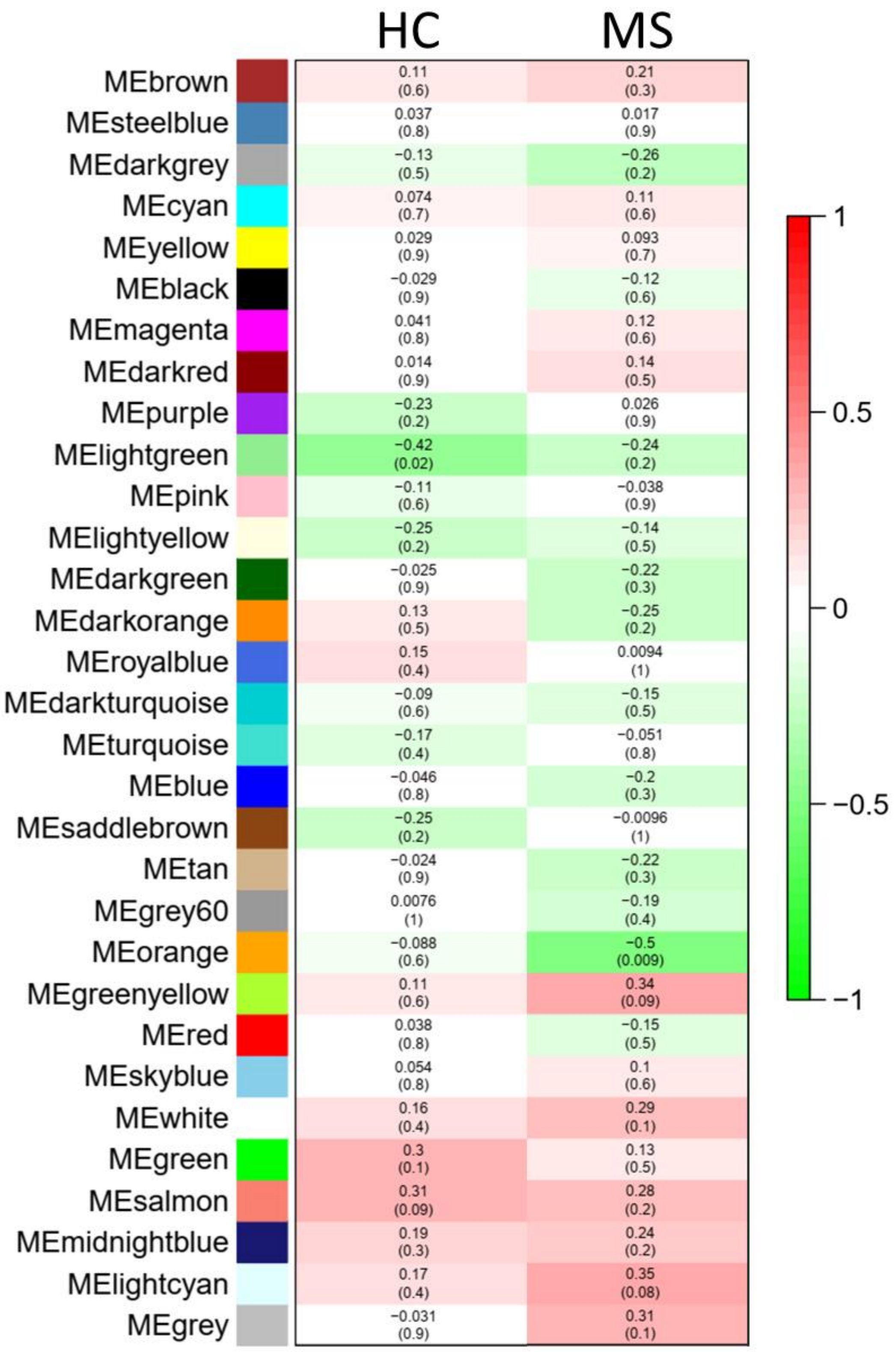

Supplementary Fig. 5: Consensus modules and correlation with vitamin D level for B cells

Consensus modules of healthy control (HC) and multiple sclerosis (MS) groups were identified. For each module, the number represents the correlation coefficient between their respective module eigengene (ME) and plasma 25(OH)D level, and the number in brackets represents the p-value.

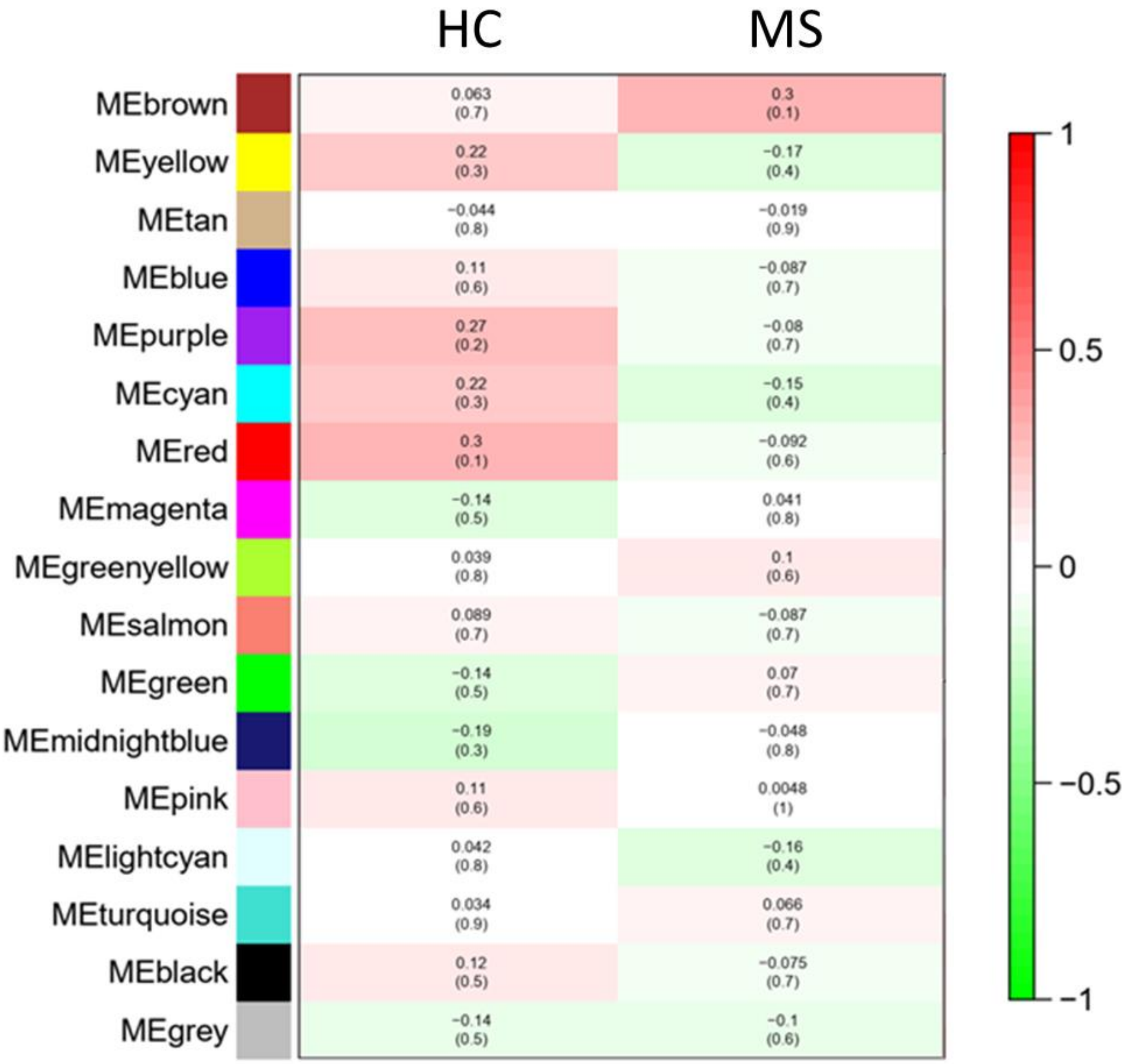

Supplementary Fig. 6: Consensus modules and correlation with vitamin D level for monocytes

Consensus modules of healthy control (HC) and multiple sclerosis (MS) groups were identified. For each module, the number represents the correlation coefficient between their respective module eigengene (ME) and plasma 25(OH)D level, and the number in brackets represents the p-value.

Supplementary Table 8: Differential expression analyses between MS cases and healthy controls of genes involved in vitamin D metabolism

Numbers in table represent log fold change (P-value) for each gene by cell type. *CYP24A1* was filtered out during pre-processing of monocyte and B cell datasets.

Gene	$CD4^+$	$CD8^+$	Monocyte	B cell
CYP24A1	-0.29 (0.37)	-0.15 (0.61)	-	-
CYP27A1	-0.00092 (1)	0.12 (0.71)	-0.25 (0.09)	0.43 (0.18)
CYP27B1	0.032 (0.82)	-0.076 (0.60)	-0.061 (0.81)	-0.25 (0.23)
CYP2R1	-0.036 (0.46)	0.0096 (0.82)	-0.017 (0.77)	0.021 (0.74)
DHCR7	-0.069 (0.18)	-0.095 (0.07)	0.00080 (0.99)	0.029 (0.64)
RXRA	0.048 (0.28)	0.033 (0.77)	0.00084 (0.98)	0.17 (0.23)
RXRB	0.047 (0.10)	-0.013 (0.68)	0.0073 (0.82)	0.0029 (0.93)
VDR	0.12 (0.09)	0.21 (0.42)	-0.043 (0.50)	-0.13 (0.51)

Supplementary table legends for supplementary table files

Supplementary Table 1: Genes whose expression were correlated with plasma 25(OH)D level for healthy controls in $CD4^+$ T cell, $CD8^+$ T cell, monocyte, and B cell subsets, respectively. Genes significantly correlated with plasma 25(OH)D was based on an unadjusted $P<0.05$.

Supplementary Table 2: **Gene set enrichment analysis (GSEA) results for genes correlated with plasma 25(OH)D level in $CD4^+$ T cells of healthy controls.** GSEA was conducted using the Molecular Signatures Database (MSigDB) and KEGG database, respectively. Pathways and gene sets were considered significantly enriched based on FDR<0.05.

Supplementary Table 3: **Gene set enrichment analysis (GSEA) results for genes correlated with plasma 25(OH)D level in $CD8^+$ T cells of healthy controls.** GSEA was conducted using the Molecular Signatures Database (MSigDB) and KEGG database, respectively. Pathways and gene sets were considered significantly enriched based on FDR<0.05.

Supplementary Table 4: Genes with differential association with plasma 25(OH)D level between multiple sclerosis (MS) cases and healthy controls in $CD4^+$ T cell, $CD8^+$ T cell, monocyte and B cell subsets, respectively ($P<0.05$).

Supplementary Table 5: Functional enrichment analysis for genes with differential association with plasma 25(OH)D level between multiple sclerosis (MS) cases and healthy controls in CD4+ T cell, CD8+ T cell, monocyte and B cell subsets, respectively. Gene sets and pathways were considered significantly enriched based on FDR<0.05.

Supplementary Table 6: Functional enrichment analysis for genes of consensus modules, as determined by weighted gene co-expression network analysis (WGCNA), which showed significant correlation with 25(OH)D level in $CD4^+$ T cells. Gene sets and pathways were considered significantly enriched based on FDR<0.05.

Supplementary Table 7: Functional enrichment analysis for genes of consensus modules, as determined by weighted gene co-expression network analysis (WGCNA), which showed significant correlation with 25(OH)D level in $CD8^+$ T cells. Gene sets and pathways were considered significantly enriched based on FDR<0.05.
